# Impacts of HIV Cure Interventions on Viral Reservoirs in Tissues

**DOI:** 10.3389/fmicb.2019.01956

**Published:** 2019-08-21

**Authors:** Paul W. Denton, Ole S. Søgaard, Martin Tolstrup

**Affiliations:** ^1^Department of Biology, University of Nebraska Omaha, Omaha, NE, United States; ^2^Institute of Clinical Medicine, Aarhus University, Aarhus, Denmark; ^3^Department of Infectious Diseases, Aarhus University Hospital, Aarhus, Denmark

**Keywords:** HIV cure, viral persistence, latency, tissues, interventional clinical trials

## Abstract

HIV reservoirs persist in infected individuals despite combination antiretroviral therapy and can be identified in secondary lymphoid tissues, in intestinal tissues, in the central nervous system as well as in blood. Clinical trials have begun to explore effects of small molecule interventions to perturb the latent viral infection, but only limited information is available regarding the impacts of HIV cure-related clinical interventions on viral reservoirs found in tissues. Of the 14 HIV cure-related clinical trials since 2012 that have evaluated the effects of small molecule interventions *in vivo*, four trials have examined the impacts of the interventions in peripheral blood as well as other tissues that harbor persistent HIV. The additional tissues examined include cerebral spinal fluid, intestines and lymph nodes. We provide a comparison contrast analyses of the data across anatomical compartments tested in these studies to reveal where peripheral blood analyses reflect outcomes in other tissues as well as where the data reveal differences between tissue outcomes. We also summarize the current knowledge on these topics and highlight key open questions that need to be addressed experimentally to move the HIV cure research field closer to the development of an intervention strategy capable of eliciting long-term antiretroviral free remission of HIV disease.

## Introduction

Despite years of successful combination antiretroviral therapy (cART), viral replication rebounds almost inevitably in all HIV infected individuals upon cART cessation (Deeks et al., 2016; Margolis et al., 2016; Wong and Yukl, 2016). This is due to the presence of persistent viral reservoirs which are the greatest barrier to an HIV cure. Because of the relative ease with which peripheral blood can be collected, this is the most common anatomical compartment analyzed in clinical HIV studies (Archin et al., 2012, 2014, 2017; Elliott et al., 2014, 2015; Rasmussen et al., 2014; Spivak et al., 2014; Mothe et al., 2015; Søgaard et al., 2015; Gutierrez et al., 2016; Leth et al., 2016; Vibholm et al., 2017, 2019a; Saxena et al., 2019; Table 1). However, it is known that HIV (and SIV in non-human primates) persists in multiple organ systems throughout the body during cART and that peripheral blood reservoir findings may not accurately reflect reservoirs in tissue (Costiniuk and Jenabian, 2014; Lamers et al., 2016; Rose et al., 2016, 2018; Estes et al., 2017; Nolan et al., 2018). Thus, it is essential that the impacts of curative strategies in all relevant organ systems be defined.

**TABLE 1:** Overview of tissue analyses in clinical studies dosing small molecule interventions in HIV cure-related context.

**Drug class**	**Drug**	**Trial identifier**	**Intervention**	**Tissues (other than peripheral blood) examined and analyses performed**	**Primary study citation**	**Sub-study citations**
Histone deacetylase inhibitors	Vorinostat	NCT01319383	Single dose of vorinostat	No other tissues examined	Archin et al., 2012	Wu et al., 2017
			22 cyclical doses of vorinostat over 12–16 weeks	No other tissues examined	Archin et al., 2014	Wu et al., 2017; Garrido et al., 2019
			Up to 10 doses of vorinostat given at 72-h intervals	No other tissues examined	Archin et al., 2017	Wu et al., 2017
		NCT01365065	Daily vorinostat for 14 days	Rectal biopsies: CA US HIV RNA^#^; HIV DNA; T cell activation	Elliott et al., 2014	Mota et al., 2017
	Panobinostat	NCT01680094	Panobinostat dosed three times per week every other week for 8 weeks	Cerebral Spinal Fluid : HIV RNA; levels of biomarkers of neurodegeneration Sigmoid Biopsies : HIV DNA; T cell activation; T cell cytokine production; virus clonality	Rasmussen et al., 2014	Christensen et al., 2015; Hogh Kolbaek Kjaer et al., 2015; Olesen et al., 2015; Rasmussen et al., 2015; Barton et al., 2016; Lee et al., 2017; Wu et al., 2017; Brinkmann et al., 2018; Garrido et al., 2019
	Romidepsin	NCT02092116	3 romidepsin infusions once weekly for 3 weeks	No other tissues examined	Søgaard et al., 2015	Jorgensen et al., 2018
			6-Vacc4x^Δ^ immunizations followed by 3 romidepsin infusions	No other tissues examined	Leth et al., 2016	Tapia et al., 2017; Jorgensen et al., 2018
Aldehyde Dehydrogenase Inhibitor	Disulfiram	NCT01286259	Daily disulfiram for 14 days	No other tissues examined	Spivak et al., 2014	None indexed in PubMed
		NCT01944371	3-day course of disulfiram	No other tissues examined	Elliott et al., 2015	Lee et al., 2019
		NCT01571466	3 immunizations of MVA-B^∗^ ± 3 months once daily disulfiram	No other tissues examined	Mothe et al., 2015	None indexed in PubMed
PKC^‡^ Agonist	Bryostatin-1	NCT02269605	Single dose of bryostatin-1	No other tissues examined	Gutierrez et al., 2016	None Indexed in Pubmed
TLR3^§^ agonist	Poly-ICLC^‡⁣‡^	NCT02071095	2 consecutive daily doses	No other tissues examined	Saxena et al., 2019	None indexed in PubMed
TLR9^§§^ Agonist	MGN1703	NCT02443935	Twice-weekly dosing for 4 weeks	Sigmoid Biopsies : HIV DNA; T cell activation; microbiome diversity; RNASeq, IHC^†^ for interferon response	Vibholm et al., 2017	Krarup et al., 2017
			Twice-weekly dosing for 24 weeks	Inguinal Lymph Nodes : CA US HIV RNA^#^; HIV DNA; immune cell activation; B cell differentiation and maturation; antibody production, glycan status and HIV-specificity; RNASeq; IHC^†^ for interferon response; IF^†⁣†^ for follicles; ISH^†⁣†⁣†^ for HIV RNA; virus clonality	Vibholm et al., 2019a	Schleimann et al., 2019; Vibholm et al., 2019b

*^#^CA US HIV RNA: Cell-associated unspliced HIV RNA; ^Δ^Vacc-4x: a synthetic p24 gag peptide vaccine; ^∗^MVA-B: modified vaccinia Ankara-based HIV-1 vaccine; ^‡^PKC: Protein kinase C; ^‡⁣‡^Poly-ICLC: Polyinosinic-polycytidylic acid, and poly-L-lysine; ^§^ TLR3: Toll-like receptor 3; ^§§^ TLR9: Toll-like receptor 9; ^†^IHC: Immunohistochemistry; ^†⁣†^IF: Immunofluorescence; ^†⁣†⁣†^ISH: *In situ* hybridization*

There are many unanswered questions regarding the impacts of HIV cure-related interventions on systemic HIV persistence. Among these are: Do findings in peripheral blood reflect outcomes in other tissues like the intestines and lymph nodes? and Do persistent viruses move freely between anatomical compartments? Studies have indicated that compartmentalization of HIV-infected cells into specific anatomical compartment and/or immunological sanctuaries occurs in untreated infections and that this compartmentalization persists during suppressive cART (Blackard, 2012). In addition, some of the drugs that have been tested in HIV cure trials may have reduced penetration into these compartments which may impede HIV cure efforts (Berg et al., 2004; Rasmussen et al., 2015). Therefore, we have focused this review on the impacts of HIV cure-related strategies onto clinical studies dosing small molecule interventions [e.g., histone deacetylase inhibitors, PKC agonist, disulfiram and *toll*-like receptor (TLR) agonists] and examining HIV persistence in tissues. Specifically, we review the impacts of such interventions on mechanisms that regulate HIV persistence *in vivo* as well as the immunological and virological impacts of these interventions in tissues other than peripheral blood. Other HIV cure related interventions including gene therapy approaches, stem cell transplants, antiretroviral intensification, therapeutic vaccines and broadly neutralizing antibody infusions have recently been reviewed elsewhere (Rasmussen and Sogaard, 2018; Caskey et al., 2019). Similarly, we callout several key animal model findings but do not elaborate upon animal model studies of HIV cure-related interventions as these data have also recently been reviewed elsewhere (Micci et al., 2015; Denton et al., 2016; Policicchio et al., 2016; Nixon et al., 2017; Honeycutt and Garcia, 2018; Whitney and Brad Jones, 2018).

## Effects of HIV Cure Interventions in the Central Nervous System, Intestines, and Lymph Nodes

We and others have made efforts to complement peripheral blood analyses by defining the effects of various HIV cure-related interventions on HIV persistence within key tissues. In these trials, the intervention were either suberoylanilide hydroxamic acid (SAHA), panobinostat or the TLR9 agonist MGN1703 (Elliott et al., 2014; Rasmussen et al., 2014; Vibholm et al., 2017, 2019a). In the trial exploring SAHA as a latency reversing agent (LRA), the Lewin group examined rectal tissue biopsies (Elliott et al., 2014). In our panobinostat trial, we examined cerebral spinal fluid and sigmoid biopsies (Christensen et al., 2015; Rasmussen et al., 2015). And in our MGN1703 trials, we examined sigmoid biopsies and lymph nodes (Krarup et al., 2017; Schleimann et al., 2019). All these anatomical reservoir studies were longitudinal in design as they included analyses of samples at baseline as well as near the end of the dosing period for the interventional drug in the respective study.

Human and animal study data highlight the potential for the central nervous system to function as an HIV reservoir or sanctuary site for the virus during treatment (Clements et al., 2005; Barber et al., 2006; Churchill et al., 2009; Zink et al., 2010; Queen et al., 2011; Gray et al., 2014; Honeycutt et al., 2017, 2018). Investigators have examined the toxicity and latency reversal effects of multiple agents including panobinostat and romidepsin on primary astrocytes *ex vivo* (Gray et al., 2016). These agents were found to be non-toxic and capable of inducing viral transcription at therapeutic concentrations. Our study provides the only published *in vivo* human data on central nervous system effects of a latency reversal agent to date (Rasmussen et al., 2015). We found that repeated, cyclic treatment with panobinostat did not lead to central nervous system adverse effects according to cerebral spinal fluid biomarkers of inflammation and neurodegeneration. We also found that panobinostat did not sufficiently penetrate the central nervous system to detectable levels and that there were no treatment-associated changes in HIV reservoir detection in the cerebral spinal fluid (Rasmussen et al., 2015). This study represents a single foray into determining the *in vivo* effects of HIV cure interventions in the cerebral spinal fluid for one intervention. However, this finding may not be specific for panobinostat since it has been shown in non-human primates that the concentration of another latency reversal agent romidepsin in cerebral spinal fluid is only approximately 2% of the level found in plasma (Berg et al., 2004). Given the scarcity of data, drawing conclusions about distinct HIV cure-related intervention impacts in the central nervous system is premature.

The role of intestines in HIV persistence has been researched extensively in humans as well as non-human primates (Anton et al., 2003; Guadalupe et al., 2003; Brenchley et al., 2004; Mehandru et al., 2004; Li et al., 2005; Mattapallil et al., 2005; van Marle et al., 2007; Chun et al., 2008; Ciccone et al., 2010; North et al., 2010; Yukl et al., 2010a, b; Chege et al., 2011; Lerner et al., 2011; Evering et al., 2012; Horiike et al., 2012; Kline et al., 2013; Deere et al., 2014; Estes et al., 2017). In a clinical study of 14 days of repeated administration of the histone deacetylase inhibitor SAHA, HIV RNA levels in rectal CD4 + T cells were modestly increased and HIV DNA levels were unchanged (Elliott et al., 2014). This outcome was also realized in our studies of the sigmoid colon during panobinostat as well as MGN1703 dosing given that we did not observe cohort wide changes in the size of the HIV reservoir in either study (Christensen et al., 2015; Krarup et al., 2017). While SAHA had no impact on T cell activation in rectal tissue, panobinostat dosing was associated with a decreased frequency of CD69+ intestinal T cells (Elliott et al., 2014; Christensen et al., 2015). This was in contrast to findings in peripheral blood where T cell activation (CD69+) was found to be increased following the first doses of panobinostat (Brinkmann et al., 2018). Additionally, we observed that panobinostat increased IL-17A expression in the intestinal epithelium and IL-17a is known to induce the production of antimicrobial peptides that may help to maintain the intestinal epithelial barrier which is damaged during HIV infection (Brenchley et al., 2006; Liang et al., 2006; Christensen et al., 2015). With dosing of the TLR9 agonist MGN1703, a robust interferon response was noted in the sigmoid colon (Krarup et al., 2017). This interferon response in the intestine was quite distinct from that observed in the peripheral blood of the same individuals (Vibholm et al., 2017). Specifically, we observed that both type I and type II interferons were generated in the periphery of treated individuals but only a type I interferon response was detected in the sigmoid colon. The type I interferon response in the colon was associated with a trend toward improved intestinal microbiome species composition. Furthermore, we found that higher baseline levels of TLR9 expression in the intestine was associated with greater reductions in levels of integrated HIV DNA during MGN1703 treatment (Krarup et al., 2017). This result suggests that tissue-specific biomarkers may help determine which individuals will exhibit the strongest response to HIV cure-related interventions in future studies. Overall, these analyses examining the intestines of participants taking HIV cure-related interventions show that the intestines are an important anatomical site for study as the peripheral blood and intestines did not always exhibit similar responses to treatment in the examined parameters. Future studies will benefit from incorporating comprehensive intestinal biopsy analyses into the study plan.

The importance of lymphoid tissues, particularly lymph nodes, in HIV persistence is clear (Shen et al., 2003; Dinoso et al., 2009; Fukazawa et al., 2015; Banga et al., 2016; Deleage et al., 2016; Lorenzo-Redondo et al., 2016; Estes et al., 2017). Beyond the observation that lymph node tissues showed no changes in SIV reservoirs in non-human primates given SAHA (Del Prete et al., 2014), there has been no published data on the impacts of HIV cure-related interventions *in vivo* in lymph node tissues. To begin addressing this major knowledge gap in the field, we undertook a longitudinal study of inguinal lymph nodes in participants taking the TLR9 agonist MGN1703 for 24 weeks (Schleimann et al., 2019). We found that lymph nodes exhibited a potent interferon response to MGN1703 dosing as was observed in peripheral blood (Vibholm et al., 2019a). We also observed similarities between the lymph nodes and peripheral blood regarding significant changes in B cell differentiation and maturation levels in response to TLR9 agonist treatment. Related to these observations, we found that MGN1703 increased plasma IgG levels as well as increased AID expression in lymph nodes (Schleimann et al., 2019). Furthermore, after 24 weeks of MGN1703 dosing, plasma and lymph node IgG glycosylation patterns were significantly altered. Changes in glycosylation were associated with reductions in viral reservoir. This study revealing similarities between the peripheral blood and lymph node responses to MGN1703 is a beginning in the process of understanding the lymphoid tissue effects of HIV cure-related interventions.

## No Evidence of Viral Compartmentalization in TLR9 Agonist Therapy Trial

While the direct *in vivo* impacts of HIV cure-related clinical interventions on latency controlling mechanisms have not been fully elucidated, there are multiple analyses that have focused on determining whether such interventions impact only clonal HIV isolates or reactivate a broad spectrum of persistent HIV isolates. These phylogenetic analyses performed with clinical trial samples have revealed that the histone deacetylase inhibitors SAHA, panobinostat and romidepsin reactivate latent viruses with unique sequence signatures as well as families of virus clones (Barton et al., 2016; Winckelmann et al., 2017, 2018). The panobinostat study also provided the first *in vivo* observation of a tissue-derived cell (i.e., a sigmoid colon lamina propria mononuclear cell) harboring an HIV provirus that matched plasma-derived rebound viruses isolated following analytical treatment interruption (Barton et al., 2016). Thus, these HIV cure-related interventions have broad latency reversing capacity *in vivo* in HIV infected individuals.

We recently examined the clonality of persistent virus in lymph nodes and compared these sequences to replication competent viruses that rebounded during an analytical treatment interruption (Vibholm et al., 2019b). We examined samples from our clinical trial in which participants received 24 weeks of repeated TLR9 agonist treatment. When we compared the latent viruses obtained from CD4+ T cells in peripheral blood and lymph nodes to viruses emerging during treatment interruption, we found there was no overlap between latent reservoir and rebound sequences. This was true even though 98% of intact or replication competent clonal sequences overlapped between these two anatomical compartments. Although rebound viruses were not derived from reservoirs detected in either blood or lymph node, we were able to show that rebound viruses were generated by recombination events between viruses within these two compartments (Vibholm et al., 2019b). This observation is consistent with peripheral blood data showing that recombination events are important during the emergence of rebound viremia (Lu et al., 2018). Whether the recombination events are due to improved viral fitness or escape of immune pressure is not yet known (Streeck et al., 2008; Ritchie et al., 2014). Overall, these data indicate that CD4+ T cells harboring latent HIV circulate between blood and lymphoid tissues during cART.

## Conclusion

Understanding the regulation of HIV reservoir persistence is a high priority in the HIV cure research field. Since 2012, 14 HIV cure-related clinical trials have been published where the objective was to test the impacts of small molecule interventions designed to either cause infected cells to become visible to the immune system for clearance or to improve the ability of the immune system to clear infected cells. These clinical studies have yielded new insights into the effects of the interventions on the regulation of HIV persistence, particularly related to clonal populations of latently infected cells. In four of the trials, efforts were made to define the impacts of the respective intervention in the central nervous system, intestinal tissues and/or lymph nodes. Data from these four trials reveal key similarities between the peripheral blood and the organs. Furthermore, they highlight that observations made in peripheral blood are not always fully representative of the impacts made by interventions in the organs which also harbor persistent HIV reservoirs. Such differential responses highlight the importance of defining the impacts of curative strategies in all relevant organ systems including those reviewed herein as well as other applicable tissues such as spleen and bone marrow. With improved methods for analyzing tissue reservoirs, investigators will begin to overcome the limitations in studies that are due to the extreme rarity and heterogeneity of HIV infected cells *in vivo* in the setting of cART. Advancement of the HIV cure research agenda will benefit from a continued push to seek detailed explorations of infected cells both derived from peripheral blood as well as from organ sources. Thus, there is strong impetus to continue examining multiple organs in such trials.

## Author Contributions

PD wrote the first draft the manuscript. OS and MT helped to wrote the manuscript. All authors read and approved the final manuscript.

## Conflict of Interest Statement

The authors declare that the research was conducted in the absence of any commercial or financial relationships that could be construed as a potential conflict of interest.
